# Promising applications of human-derived saliva biomarker testing in clinical diagnostics

**DOI:** 10.1038/s41368-022-00209-w

**Published:** 2023-01-04

**Authors:** Mengyuan Song, Hao Bai, Ping Zhang, Xuedong Zhou, Binwu Ying

**Affiliations:** 1 Department of Laboratory Medicine, West China Hospital, Sichuan University, Chengdu, China; 2 State Key Laboratory of Oral Diseases & Human Saliva Laboratory & National Clinical Research Center for Oral Diseases & West China Hospital of Stomatology, Sichuan University, Chengdu, China

**Keywords:** Diagnostic markers, Molecular medicine

## Abstract

Saliva testing is a vital method for clinical applications, for its noninvasive features, richness in substances, and the huge amount. Due to its direct anatomical connection with oral, digestive, and endocrine systems, clinical usage of saliva testing for these diseases is promising. Furthermore, for other diseases that seeming to have no correlations with saliva, such as neurodegenerative diseases and psychological diseases, researchers also reckon saliva informative. Tremendous papers are being produced in this field. Updated summaries of recent literature give newcomers a shortcut to have a grasp of this topic. Here, we focused on recent research about saliva biomarkers that are derived from humans, not from other organisms. The review mostly addresses the proceedings from 2016 to 2022, to shed light on the promising usage of saliva testing in clinical diagnostics. We recap the recent advances following the category of different types of biomarkers, such as intracellular DNA, RNA, proteins and intercellular exosomes, cell-free DNA, to give a comprehensive impression of saliva biomarker testing.

## Introduction

High accessibility of saliva makes it the center of biomarker research. Especially during the pandemic, saliva has been regarded as a useful and cost-effective tool for coronavirus disease 2019 (COVID-19).^1,2^ The detection limit of the viral genome enables it a precise method, with the detection sensitivity ranging from below 100 copies/mL of the virus to 0.38 copies/μL. Coincidentally, human papillomavirus (HPV) genes or somatic variants of head and neck squamous cell carcinomas (HNSCC) were detected more frequently in saliva than that in plasma, indicating that saliva appeared to be a meaningful way to identify HNSCC.^3^ Accordingly, there are some breakthroughs in saliva testing, such as human immunodeficiency virus (HIV) testing as an indicative factor for acquired immunodeficiency syndrome (AIDS), HPV detection for HNSCC. Besides, myocardial enzyme testing for the monitoring of myocardial infarction and cortisol for stress and inflammation status identification also remind us of the application of saliva in human-derived biomarker discovery. Saliva testing shows its diagnostic value in oral diseases, diabetes, renal diseases, hepatitis, neurodegenerative diseases, and immunodeficiency disease. These breakthroughs mostly rely on the good correlation of the biomarker concentration between saliva and blood. For example, a previous article showed significant correlations of free cortisol levels in saliva and blood, thus enabling the saliva cortisol testing to show psychobiological states.^4^

**Fig. 1:**
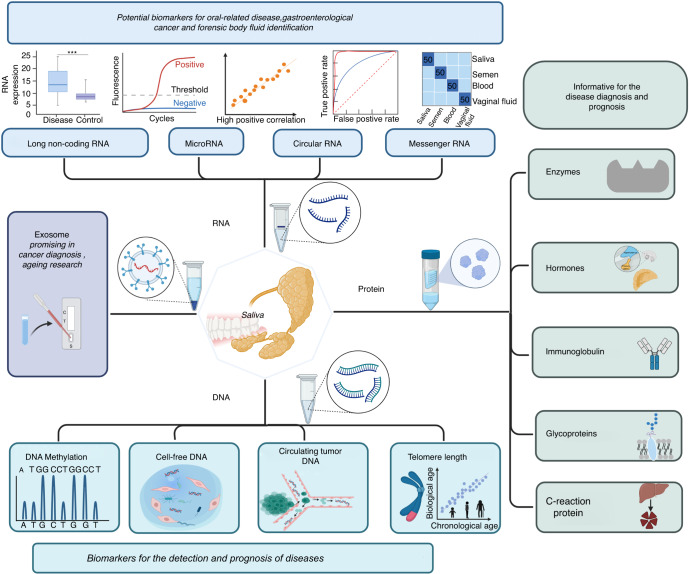
The scheme of the review organized by different types of biomarkers. Some categories were listed specifically, such as C-reaction protein, because the research is relatively dominant. The figure is created with BioRender.com

Interestingly, a study showed that people who shared saliva, such as drinking juice with the same straw, had more intimacy with each other.^5^ On the other hand, women abused by their intimate partner bear detectable cortisol level change in saliva.^6^ This shows the informativeness of saliva in psychological research. These studies extend our basic knowledge about saliva. Saliva is growing more important to health issues. Salivary gland organoid culture also became possible, which promotes the study of salivary diseases.^7^ Non-human derived biomarkers, such as oral microbiota, was thoroughly commented in another review.^8^ The review summarized oral microbes in many situations, such as digestive diseases, cancers, and cardiovascular diseases. It highlights the systematic interaction of oral microbes and humans. In addition, a review published in 2016 has summarized the diagnostic value of saliva in diseases.^9^ It mainly addresses the proceedings from 2016 to 2022, to shed light on the recent promising usage of saliva testing in clinical diagnostics. Here in this review, we made an update of saliva testing in the framework of major biomarker types, as shown in Fig. 1. Articles whose keywords contain “human”, “saliva” and the corresponding biomarker name were extracted from Web of Science and taken into consideration.

### Secreting mechanisms of saliva

Whole saliva is a complex mixed fluid containing saliva, gingival crevicular fluid, epithelial cells, microorganisms, and food debris.^10^ The fraction of substances in the whole saliva is dynamic and affected by various stimuli. In general, a healthy adult daily produces 500–1 500 mL of the whole saliva at an average speed of 0.3–0.4 mL per min.^10,11^ Salivary glands are the main producers of saliva, which contain three pairs of major salivary glands (parotid, submandibular and sublingual gland) and 600 to 1 000 minor salivary glands. Acinar cells are the basic units of salivary glands secrete the mixed fluid, which can be categorized into two types: serous and mucous cells. The parotid glands have plasmacytoid acinar cells and secrete thin, watery and amylase-rich saliva. The sublingual and submandibular glands are mixed glands, consisting of serous and mucous cells that secrete a mucus-rich fluid. The mixed fluid produced from acinar cell enters the collecting ducts for reabsorption. During this process, the composition of the secretion changes and eventually leads into the oral cavity along the single large ducts of the salivary glands. The main excretory ducts of parotid glands open onto the oral mucosa near the upper molars. The dominant duct of the submandibular gland and the sublingual gland leads to the sublingual mucosa of the mouth floor. The primary source of the initial fluid secreted by the salivary glands is the intercellular fluid, while the final mixed fluid flowing into the oral cavity is mainly derived from intercellular fluid and blood.^12^ Thus, various substances in blood also can be detected in saliva. Salivary glands are controlled by autonomic nervous system and regulated by the reflexes. Stimuli such as chewing, taste, smell, sight, and pain can cause salivary secretion.^13^ Pathologies of the salivary glands, oral tissues and other organs can lead to abnormalities of salivary secretion, causing volume and composition changes. It facilitates research into the diagnosis, treatment and prognosis of diseases using biomarkers in saliva.

### The substances in saliva and other common body fluids

There are 99% water, 0.5% organic, inorganic matter, and numerous cellular elements in the whole saliva.^14,15^ The main prevalent organic substance is composed of enzymes such as amylases, lysozyme, peroxidase, lipase, hormones such as cortisol, cytokines, immunoglobulins, mucins, lactoferrins, etc. While potassium, sodium, chloride, calcium, magnesium, and bicarbonate are the customary inorganic components of saliva.^15^ Saliva plays a vital role in oral health and general health. It is these salivary substances that maintain the physiological homeostasis of the oral cavity. The major component, water, moistening the surface of the oral cavity, make saliva texture stickiness and perform an oral clearance. Electrolytes can neutralize acid and act as a buffer (bicarbonate) and are responsible for tooth protection by preventing demineralization and enhancing remineralization of enamel (calcium, fluoride, phosphate, and bicarbonate).^16^ The functions of organic substances include antimicrobial, tissue repair, digestion, and formation of salivary sacs. For instance, lysozyme can hydrolyze the cell wall of Gram-positive bacteria and destroy the structure of bacteria, while immunoglobulin (e.g., sIgA) inhibits microbial adhesion and reduces microbial reproduction. Mucin protects the oropharyngeal mucosa from injury.^10^ The cellular elements in saliva contained leukocytes, erythrocytes, desquamated oral epithelial cells, microbial cells, and products of cells and microorganisms. The newly identified tumor markers encapsulated in exosomes, have also been found stably exist in saliva.^17,18^ The composition of and proportion of substances in saliva are dynamic with physiological state. Disease, diet, and mental status can cause changes in saliva composition. The disease-related change of salivary substance facilitates the application of saliva in clinical research, such as the diagnosis, therapy, and prognosis.

Among all the body fluids, sweat is very similar as saliva and is also easily accessed. Whereas, saliva is quite different from sweat, drops secreted through the pores of the skin. It is believed that there are no cells in sweat, while in saliva there are many cells. Instead, trace minerals, lactic acid, and urea appear in sweat.^19^ Some scholars discussed next-generation digital based biomarkers for tuberculosis diagnosis and therapeutic drug monitoring approach, both in sweat and saliva.^20^ Recently, studies about sweat distributed in wearable devices or detecting biosensors.^21,22^ Ray et al. developed a label-free optical detection system to simultaneously examine multiple stress biomarkers.^23^ They found that cortisol could be detected in sweat, saliva, urine, plasma, and serum; serotonin and dopamine were measurable in plasma and serum; neuropeptide Y was detected in all these four biological fluids. The composition of seven kinds of common body fluids was listed in Table 1.

**Table 1:** The composition of different body fluids

Type	pH	Specific gravity	Major component	Minor component	Volume	Difficulty of accessibility
Saliva	6.6–7.1	1.002–1.008	>99% H_2_O	Electrolytes, immunoglobulins, proteins, enzymes, mucins	0.5–1.5 L	⭐
Plasma	7.35–7.45	1.025–1.030	>90% H_2_O	Ions, proteins, dissolved gases, nutrient molecules, and wastes	2.75–3.30 L	⭐⭐⭐
Urine	4.5–8.0	1.010–1.030	95% H_2_O	Urea (2%), creatinine (0.1%), uric acid (0.03%)	0.8–2.0 L	⭐⭐
Sweat	4.0–6.8	1.004–1.011	>99% H_2_O	NaCl (mostly) and other electrolytes	0.6–0.7 L (unconscious)	⭐⭐
Tear fluid	5.20∼8.35	1.008	98% H_2_O	Electrolytes, proteins, lipids, mucins	NA	⭐⭐
Breast milk	6.35–7.35	1.030	87% H_2_O	1% protein, 4% lipid, and 7% carbohydrate	0.75–1.035 L	⭐⭐⭐
Cerebrospinal fluid	7.334–7.523	1.006–1.008	99% H_2_O	Electrolytes, proteins, glucose	0.1–0.15 L	⭐⭐⭐⭐⭐

### Promising saliva DNA biomarkers

Literature searching results for salivary DNA biomarkers mainly fall into four topics, which are DNA methylation, telomere length, cell-free DNA, and circulating tumor DNA. Among these four topics, DNA methylation studies account for the most part.

### DNA methylation

Most epigenetic studies used blood samples for biomarker selection.^24^ Before 2016, published studies about saliva methylation largely focused on cancer, psychiatry, environmental, and lifestyle related disease.^25^ In the past five years, saliva methylation studies have mainly focused on the alteration caused by environmental exposure, such as air pollution and smoking. Bakulski et al. investigated the effect of environmental exposure, such as PM2.5 and PM10, on saliva DNA methylation signatures.^26^ They tested DNA methylation levels in saliva at ages 9 and 15 for association with PM10 exposure at birth. Dawes et al. studied DNA methylation level at cg05575921 using saliva samples from high school students. Their paired serum cotinine (a key nicotine metabolite) values were accessible. The result indicated that saliva DNA methylation held promise for the detection of nascent smoking.^27^ They did not examine if the marker could predict smoking tendencies. On the other hand, another study indicated that methylation in the tumor suppressor genes p16 and p14 was not detected in saliva from nontobacco users, while partial methylation was detected in 33.3 % samples of smokeless tobacco users.^28^ Saliva methylation also has the potential in identifying smokers and non-smokers, by detecting methylation status at cg05575921.^29^ The research showed that smoking induced a demethylation response. Another research also investigated the correlation between smoking and salivary cg05575921 methylation.^30^

Using saliva DNA can be problematic since it mixes human and bacterial DNA. A high proportion of bacterial DNA may bring possible failure in genetic tests.^25,31^ Saliva from the same individual at different time points may differ widely regarding cell composition.^32^ Some scholars constructed a salivary DNA methylation reference panel for individuals aged from 7 to 16 years old, estimating cell-type heterogeneity within the same individual.^33^ Rounge et al. concluded an association between the saliva methylome and body-mass index (BMI) in adolescence.^34^ Moccia et al. showed that DNA methylation patterns differed in cord blood and infant saliva,^35^ leading to distinct birthweight signatures. Another study reported that three obesity-related outcomes were negatively associated with LEP gene methylation in obese boys only.^36^ A high correlation between blood and saliva methylation levels in respiratory allergy individuals also gave credit to the usefulness of saliva in epigenetic studies.^37^

In oral diseases, Mimansha et al. detected promoter methylation in tobacco users with and without oral typical premalignant lesions It concluded that promoter methylation levels of various genes were linked to a high probability to precede oral squamous cell carcinomas.^38^ A significant hypermethylation of p16 appeared in buccal cells and saliva of oral submucous fibrosis patients.^39^ Saliva cells of smokers with chronic periodontitis tend to have a methylated SOCS1 gene promoter rather than saliva cells from the non-smoking patients, as was reported by Martinez et al.^40^ Yamaguchi et al. found significant methylation alterations in 2381 CpG sites after orthognathic surgery. It was perhaps due to the response to surgical stress or bone injury.^41^

In neurodegenerative diseases, Chuang et al. unveiled five significant CpGs.^42^ Elaheh et al. observed allele-specific methylation in CD4^+^ cells instead of saliva.^43^ In autoimmune diseases, altered DNA methylation profiles appear to be present in saliva in Coeliac disease individuals.^44^ Biomarker research boom in neurodegenerative diseases might be due to the difficulty in the obtaining of the brain for biopsy. In psychiatric disorders, saliva BDNF gene methylation is useful for treatment monitoring of borderline personality disorder (BPD).^45^ Psychotherapeutic intervention can significantly lower BDNF methylation in BPD patients’ saliva samples. In blood, this tendency did not exist. In attention-deficit/hyperactivity disorder, children under this disorder were tested with altered VIPR2 gene methylation signatures.^46^ Additionally, there are other applications of salivary epigenetic biomarkers. Hearn et al. showed a high correlation coefficient ( r= 0.92 ~ 0.95, *P* < 0.001) between saliva and intestinal mucosa.^47^ This is valuable for the monitoring of intestinal diseases, with the convenience of multiple testing.

In forensic science, saliva methylation is also a heavily watched biomarker for the age estimation of biological samples. Hong et al. used a panel of 7 CpG markers to construct an age prediction panel with high predictability.^48^ They built a model with a mean absolute deviation (MAD) of 3.13 years. Lee et al. applied the droplet digital PCR (ddPCR) assays to study DNA methylation level, resulting in an MAD of 3.3 years.^49^ Body fluid discrimination is also a frequently studied question. Using methylation can distinguish different kinds of biological fluids or perform age prediction in sperm, saliva, blood, and menstrual blood.^50–56^ Age prediction by saliva methylation detection in cigarette butts is also widely used in forensic investigations.^57^ Interestingly, interethnic methylation differences were observed in Japanese and Indonesian populations.^58^

Illumina Methylation 450K BeadChip platform was most widely used (Table 2). Additionally, methylated DNA immunoprecipitation coupled to next-generation sequencing (MeDIP-seq) was also explored in detecting saliva methylation.^59^ These were high throughput detecting assays. Pyrosequencing, methylation-specific polymerase chain reaction and agarose gel electrophoresis and multiplex methylation SNaPshot reactions were preferred in target methylation site testing.

**Table 2:** The methods and marker sets used in saliva methylation studies from 2016 to 2022

Method used for methylation detection	Marker sets	References
1. MALDI-TOF mass spectrometer	27 CpG sites in the promoter (MT2 region)	^25^
2. Infinium HumanMethylation450K array	485 000 probes	^26,35,37,41,42,44,46,47,51,301^
3. ddPCR	(1) cg05575921 and DMR16(	^27,29,30^
	2) cg14361627, cg14361627, cg08928145 and cg07547549	^302^
4. Illumina MethylationEPIC BeadChip	795,694 probes	^33,54^
5. methylation specific polymerase chain reaction and agarose gel electrophoresis	(1) p16INK4a and p14ARF	^28^
	(2) SOCS1	^40^
	(3) p16, DAP- K, MGMT, and GSTP1	^38^
	(4) ZNF282 and HPCAL1	^52^
6. targeted bisulfite sequencing (TBS) protocol	3.1 million CpG sites	^34^
7. pyrosequencing	(1) LEP, ICAM-1, CRH, and LINE-1 genes	^36^
	(2) BDNF	^45^
	(3) NMUR2 and UBE2U	^50^
	(4) ZC3H12D, BCAS4 and cg06379435	^53^
	(5) FADS1 and FADS2	^43^
8. real-time methylation-specific polymerase chain reaction	(1) P16	^39^
	(2) VM03 and SM02	^55^
9. multiplex methylation SNaPshot reactions	(1) cg00481951, cg19671120, cg14361627, cg08928145, cg12757011, cg18384097 and cg07547549	^48,51^
	(2) ELOVL2, FHL2, KLF14, C1orf132/MIR29B2C, and TRIM59 genes	^56^
10. methylation-sensitive high-resolution melting (MSHRM)	(1) ELOVL2 and EDARADD	^57^
	(2) EDARADD and FHL2	^58^
11. methylated DNA immunoprecipitation coupled to next-generation sequencing (MeDIP-seq)	Genome-wide	^59^

### Telomere length

Matthew et al. investigated telomere length in saliva samples of healthy athletes.^60^ They showed that samples collected pre-season had shorter telomeres than those collected mid-season. Also, males had longer telomeres than females. More than a decade ago, Lahnert reported an improved method for detecting saliva telomere length for its use in the inference of age.^61^ It showed that the precision of the measurements in saliva specimens was lower than that in blood. Another research showed that telomere length was longer in saliva than in blood.^62^

### Cell free DNA (cfDNA)

After apoptosis or necrosis, cell debris including nucleotides releases into the interstitial fluid. Thus, individuals with a high rate of cell death tend to have a higher proportion of cfDNA. Interestingly, psychosocial stress exposure can contribute to a higher level of cfDNA release. The correlation between salivary cortisol and cfDNA increase was observed.^63^ Additionally, saliva parasite-derived cfDNA can also be indicative of the disease.^64^

### Circulating tumor DNA (ctDNA)

While most of cfDNA comes from normal cells of the body, ctDNA is related to tumors. Salivary ctDNA mutations were found to be more frequent in oral cancers than in oropharynx, larynx, and hypopharynx cancers.^65^ Researchers focused mainly on TP53, CDKN2A, PIK3CA, FAT1, and NOTCH1 genes. It was believed that in liquid biopsy, saliva was superior to plasma in oral cancers, because of the physical location. Besides, salivary ctDNA was investigated in Non-Small Cell Lung Cancer (NSCLC). Li et al. noticed that EGFR mutation length fragments were detected with a size range below 80 bp.^66^ Epidermal growth factor receptor (EGFR) gene mutations in saliva-derived ctDNA were detected and were consistent with that of plasma ctDNA.^67^ They could not be applied in the diagnosis of NSCLC. Interestingly, Eun et al. built a mouse model to compare the correlation between the human long interspersed element (hLINE) in mouse saliva or plasma, as an indicator of ctDNA amount with metastasis or recurrence rate in orthotopic head and neck cancer (HNC).^68^ HPV-associated oropharyngeal cancer (OPC) diagnosis can also benefit from salivary cfDNA detection.^69^

### RNA biomarkers

To give a bigger scope of the focus of studies, we primarily searched the database with keywords “saliva” and “RNA”. A knowledge map was drawn to present the research focus on salivary RNA in the past six years, using R language (https://www.R-project.org/). From Supplementary Figure 1a, five main topics have been considered to study salivary RNA molecules. Understandably, “expression” was the most frequent keyword, as can be seen in Supplementary Figure 1b. Many studies aimed at discovering biomarkers for diagnosis and prognosis, although we did not include the search keyword “biomarkers” for generating this plot. Among all diseases, cancer research ranked at the top of the list. Specifically, squamous cell carcinomas were paid the most attention to. It was consistent with the result of saliva DNA biomarkers. This might be due to the direct relation between epithelial cells in saliva and these cancer types. Scholars from all over the world have been working in this research field, with or without collaborations (Supplementary Fig. 1c). These papers also mentioned serum, possibly for paired studies (Supplementary Fig. 1d, a). In the next paragraph, we discussed the papers searching by the keywords “saliva”, “RNA”, and “biomarkers”.

### mRNA

Messenger RNAs (mRNAs) are transcribed from genes to the cytoplasm and guide protein synthesis. As early as in 2004, Li et al. demonstrated that there were approximately 3 000 different mRNAs in cell-free saliva of healthy subjects, and almost 200 of these were present in all subjects.^70^ Informative cell-free human mRNAs existed in saliva. The salivary transcriptome sequencing has been proposed as a novel clinical approach to salivary diagnostics. Compared with proteomic constituents, salivary transcriptomic biomarkers moved the detection time to an earlier stage of the disease.^71^ In recent years, published studies about saliva mRNAs markers largely focused on cancers, forensics, Sjögren’s syndrome (SS), and periodontitis, as well as exploration of other diseases.

Accumulated evidence has proven the associations between saliva mRNAs and cancers.^72^ Li et al. firstly reported seven upregulated mRNAs in the saliva of oral squamous cell carcinoma (OSCC) patients, which have been independently confirmed by multicenter cohorts.^73,74^ A more recent study demonstrated a good predictive probability of 80% by four biomarkers for patients with OSCC, but not for patients with oral leukoplakia and dysplasia. Moreover, the combination of SAT and IL-8 achieved a high predictive ability of 75.5%, suggesting an important value of the two biomarkers for the early detection of OSCC.^75^ Recently, researchers have paid attention to the influence of reference gene stability and oral health condition on the performance of saliva mRNA biomarkers for OSCC.^76–78^ Martin validated the expression stability of five housekeeping genes for use in oral cancer salivary mRNA panels. Six pre-specified OSCC-associated genes were verified to be significantly upregulated in OSCC patients based on delta Cq values.^76^ Cheng et al. reported chronic periodontitis could apparently change the levels of those potential oral cancer salivary mRNAs except for S100P mRNA.^77^ Another independent study also suggested that oral inflammatory processes can have influence on the performance of the seven putative salivary mRNA biomarkers.^78^ Therefore, inflammatory biomarkers such as salivary IL6 mRNA should be utilized in the clinical application of OSCC detection.

Salivary mRNA biomarkers of other cancers have been little explored. Li et al. screened 30 salivary mRNA candidates from the salivary transcription profiles of 63 gastric cancer (GC) and 31 non-GC patients. Nine of the 30 biomarkers were verified in an independent prospective cohort. In addition, the panel of three mRNAs and two microRNAs screened by the LASSO algorithm generated the best area under curves (AUC) value of 0.81.^79^ Xu et al. subsequently showed that the expression of four mRNA biomarkers in saliva were significantly downregulated in GC patients compared to healthy controls.^80^ Besides, salivary mRNA biomarkers has been explored in breast cancer,^81^ ovarian cancer,^82^ lung cancer,^83^ and chronic myeloid leukemia.^84^ Most of those studies indicated that combined analysis of salivary mRNA biomarkers and other known biomarkers could improve the performance of cancer detection. Even so, the clinical utility of those salivary mRNA biomarkers should be further evaluated in more populations.

In forensic science, mRNA profiling for saliva identification have been evaluated by the European DNA Profiling Group (EDNAP) in the last decade. The mRNA profiling could be used for current saliva identification procedures, based on its advantages of higher sensitivity, greater specificity, multiplex detection, and compatibility with current identification method.^85,86^ Previous studies reported several salivary mRNA biomarkers including HTN3, STATH, MUC7, PRB1, PRB2, and PRB3. The first three biomarkers were evaluated by the EDNAP.^85^ In recent years, the sensitivity and specificity of those salivary mRNA biomarkers have been investigated. Three of the saliva markers showed high specificity in the discrimination of saliva and other biofluid samples including blood, semen, urine, and vaginal secretions.^87^ Among them, HTN3 was the most specific saliva biomarker for it was neither detected in nasal mucosa nor trachea samples.^88,89^ Liu et al. reported that the sensitivity of saliva mRNA using multiplex real-time PCR (RT-PCR) assay.^87^ In the routine forensic casework with the ParaDNA® Body Fluid ID System, the positive rate of HTN3 mRNA marker in saliva samples ranged from 76.9% to 95%.^90,91^ Moreover, the stability of the mRNA markers has been investigated recently. Watanabe et al. demonstrated that exposure to blue light (450 nm) had no effect on the stability of the mRNA markers.^86^

There are also significant changes in the salivary transcriptome of Sjögren’s syndrome patients. Hu et al. successfully identified 3 mRNA biomarkers promising for distinguishing primary SS.^92^ A recent study showed significantly lower mRNA levels of saliva IL-38 and IL-36R in primary Sjögren’s syndrome (pSS) group. Further experiment showed that IL-38 inhibited pSS possibly through Th17 inflammation.^93^ In recent years, increased mRNA markers such as NGAL and p16 mRNA levels were reported in salivary glands of pSS, but their expressions in saliva remained unexplored.^94,95^

In periodontal diseases, several dysregulated immune response related genes have been detected in saliva. Swaminathan et al. found that TLR2 and TLR4 mRNAs were up regulated in epithelial cells of unstimulated whole saliva from chronic periodontitis, while PGRP3 and PGRP4 mRNAs were reduced.^96^ In their further study, they confirmed that the TLR4 mRNA was significantly higher in the salivary epithelial cells of the gingivitis and the chronic periodontitis cohort as compared to that from the healthy saliva samples. It could be regarded as clinically relevant markers of disease progression from gingivitis to periodontitis.^97^ A recent study showed that MIP-1α, MMP-2 and MMP-9 mRNA expression was higher in patients with periodontal disease than in healthy individuals. These biomarkers were correlated with the severity of the disease.^98^ In addition, Detzen et al. reported the expression of CD163 as a potential salivary biomarker of periodontitis.^99^

Salivary mRNA biomarkers of other diseases have been little explored. Fagin et al. found that BZLF1 mRNA expression correlated with Epstein-Barr virus load in saliva samples, thus indicating active replication of Epstein-Barr virus.^100^ Angelova and colleagues detected the salivary mRNA levels of IL-6, MMP-8, and GSS in 28 pyelonephritis children, and demonstrated 64, 3.27, and 1.94 times increase, respectively.^101^ Fan et al. revealed reduced expression of salivary PARK2 in manganese-exposed smelting workers, which may partly explain the manganese-induced Parkinsonian disorder.^102^ Furthermore, the possibility to use those altered mRNA expressions in saliva as biomarkers for disease diagnosis deserves verification in a larger independent population.

### microRNA

MicroRNAs (miRNAs) are a set of small non-coding RNA (sncRNA) molecules that present in saliva stably because of being wrapped in lipoprotein vesicles that protected them from degradation.^103^ Before 2016, microRNAs in saliva have been studied in a variety of oral-related diseases and gastroenterological cancer. In the past five years, oral-related diseases and gastroenterological cancer have still been the research hotspot for microRNAs in saliva. Many studies have found potential biomarkers for these diseases, as shown in Supplementary table 1.^104–111^ Otherwise, other diseases and fields have also begun to pay attention to microRNAs in saliva involved in nervous system diseases, immune system diseases, infectious diseases, and forensics.

In neurological diseases, microRNA in saliva has the potential as a biomarker for autism spectrum disorder, alcohol dependence, and concussion. Several researchers had built diagnostic panels for autism spectrum disorder with a sensitivity of 81.0–93.1% and a specificity of 32.0%–87.5%.^104–106^ Otherwise, microRNA in saliva can be a potential, non-invasive biomarker using for the monitoring of alcohol abuse.^107,112^ For concussion, a panel of 14 sncRNAs could distinguish concussed subjects immediately after the game (AUC 0.91, 95% confidential interval (CI) 0.81–1) and 36–48 hours later (AUC 0.94, 95% CI 0.86 to 1).^108^ In immune system diseases, the set of miR-17-5p and let-7i-5p in saliva showed capability for differentiating patients with pSS from controls. It only needed 100 µL saliva using for microRNA extraction and isolation.^109^ In infectious disease, research focused on the diagnosis of Hand, Foot, and Mouth Disease (HFMD) resulted in 77.08%-92.86% accuracy with the 6-miRNA panel and 68.75%-91.67% with the 4-miRNA panel. sa-miR-221 was found to be consistently and significantly downregulated in HFMD cohorts.^110^ In forensics, research focuses on microRNA stability in saliva and methodology .^113^ Tiffany et al. found that saliva samples are more susceptible to environmental factors relative to blood samples.^114^

Over the years, many studies have investigated microRNAs as disease biomarkers. Research hotspots mainly focus on miR-21, miR-23, miR-1246 and so on. In recent years, some studies have also used the combination of multiple microRNAs as diagnostic markers of diseases, which can improve the diagnostic efficiency to a certain extent.

### Circular RNA

Circular RNAs (circRNAs), a type of non-coding endogenous RNA molecules, are highly stable, mostly located in cell membranes, which can modulate gene expression by interacting with proteins or microRNAs. CircRNAs participate in many biological processes. Dysregulated expression of circRNAs leads to abnormal cellular function and growth defects that are associated with disease and cancer.^115–117^ Bahn et al. validated the existence of salivary circRNAs for the first time in 2015. The circRNAs in saliva may be engaged in intercellular signaling pathways and inflammatory responses.^118^ The origin of salivary circRNAs may be the products derived from the lysis of oral cells (epithelial cells, leukocytes, and erythrocytes) and oral microorganisms, the secretion of secretory cells, and the exosomes.

CircRNAs have been demonstrated to be responsible for the proceeding of some diseases such as cardiovascular disease, diabetes mellitus, chronic inflammatory diseases, neurological disorders, and cancer.^119–121^ Zhao et al. found three upregulated circRNAs in the OSCC group. Two of were associated with TNM stage and were the potential biomarkers for diagnosing OSCC.^122^ A meta-analysis concluded a high accuracy of circRNAs in the diagnosis of OSCC.^123^ Besides, they found the diagnostic value of circRNAs in saliva was higher than that in tissues. However, this phenomenon may be caused by the unbalanced inclusion of studies. Most articles included tissue samples and only one trial obtained saliva from OSCC patients. According to the meta-analysis, it was found that the clinical application of salivary circRNAs for OSCC was less explored. The association between circRNA and disease has been widely explored. However, they mainly target the tissue and plasma. The simple, rapid, and non-invasive sampling methods and the fact that most substances in peripheral blood also exist in saliva make saliva an ideal biological fluid for disease research. As a result, salivary circRNAs need to be further studied. It is expected that as more circRNAs in saliva are characterized, and the targets are identified, they would be routinely applied to personalized medicine.

Due to tissue-specific and even cell type-specific characteristics,^124^ salivary circRNAs are also employed as biomarkers in forensic body fluid identification. In forensic identification, mRNAs are widely explored for its potential in distinguishing body fluid types. Given the high stability of circRNAs, they have the potential application to deal with samples of low quality and limited quantity. Song et al. explored microarray expression profiles of circRNAs by which body fluids from semen, venous blood, and saliva could be discriminated. It indicated that circRNAs might be potential biomarkers for forensic body fluid identification.^125^ The expression of fourteen circRNAs from five human body fluids had been investigated by Liu et al. Incorporating circRNAs into mRNA profiling improved the identification of body fluids and contributed to the establishment of a robust multiplex assay.^126^ Subsequently, the multiplex assays had been constructed to distinguish forensic body fluids.^127^ In brief, circRNAs facilitate the identification of biological origin in forensic practice, especially the degraded and aged samples.

### lncRNA

Long non-coding RNAs (lncRNAs) are non-coding RNAs more than 200 nucleotides and are important regulators of cellular processes. Detection of lncRNAs in saliva benefit for cancer diagnosis and prognosis. Tang et al. investigated the relative abundance of six well-documented cancer-associated lncRNAs and found a detectable amount of some lncRNAs in saliva. Among them, lncRNA MALAT1 existed in all the involved patients, indicating the potential of salivary lncRNAs as oral cancer biomarkers.^128^ In pancreatic cancer patients, salivary lncRNA HOTAIR and PVT1 levels were significantly higher than in benign pancreatic tumor patients and healthy controls. Further research found that the expression of salivary HOTAIR and PVT1 was pancreatic cancer-specific and significantly reduced after the curative pancreatectomy.^129^ Similarly, lnc-PCDH9–13:1 was significantly elevated in cancer tissues, plasma and saliva of hepatocellular carcinoma patients compared with healthy controls. Its abundance was largely reduced after curative hepatectomy but apparently elevated again if recurrence occurred. Possible mechanism lies in that the overexpression of lnc-PCDH9–13:1 could promote cell proliferation and migration in vitro.^130^ Shieh et al. found that people lacking salivary lncRNA X-inactive specific transcript (XIST) expression had a significantly increased risk of OSCC (AUC = 0.73).^131^

Although considered to be cancer-specific, lncRNAs are not that specific in noncancerous diseases.^132^ For instance, two cancer-associated lncRNAs, lncRNAs NEAT1 and MALAT1, were upregulated not only in cancer but also during noncancerous conditions such as SARS-CoV-2 infection.^133^ Thus, before the usage of salivary lncRNA detection in clinical applications, those biomarkers described above still require more validation studies with larger sample sizes in multi-centers.

### Protein and hormone biomarkers

Human saliva harbors a diverse repertoire of proteins and peptides that can be informative for disease diagnosis and prognosis. To date, more than 3 000 different proteins have been identified in human saliva using proteomic approaches.^134^ The identified protein species are varying from high molecular weight proteins such as glycoproteins, to small molecular weight proteins or peptides. Saliva is identified as a functional equivalent to serum. For example, one proteomic study found 1939 proteins in saliva, with 597 proteins that have been observed in plasma.^135^ The proteins and peptides in the saliva not only play important roles in maintaining oral health but may also be used as biological markers to survey health and other disease status. Chu et al. identified three proteins as potential biomarkers of oral cancer.^136^ Studies found that the saliva of patients with Parkinson’s disease (PD) was different in composition from that of healthy controls.^137,138^ Besides, human salivary glands harbor many peptides and protein-structured hormones. Salivary hormones used in clinical research have recently drawn much attention because it offers a non-invasive alternative to the test of plasma or serum hormones. This section was structured by different types of proteins and hormones. It is noteworthy that a few non-protein hormones were also included in this section.

### Enzymes

Eating is triggered by the release of saliva. Saliva is important for food digestion and energy obtaining for its multiple enzymes. Enzymes in saliva include salivary amylase (also known as ptyalin),^139,140^ lysozyme, lingual lipase, carbonic anhydrase, maltase, phosphatase, carbonic anhydrase, kallikrein, and peroxidases.^141–143^ Many of the enzymes have been utilized as diagnostic markers. Approaches based on salivary enzymatic methodologies are widely used for the diagnosis of diseases, including cardiovascular diseases,^144^ Alzheimer’s disease,^145^ oral diseases,^146,147^ and cancer.^148^ Two major focuses of salivary enzymes were pepsin and amylase.

Pepsin is the proteolytic enzyme of pepsinogen and is activated by hydrochloric acid, which has a hydrolytic effect on proteins. It is released solely by gastric chief cells and participate in the development of reflux-related disorders. It can be detected in saliva when gastroesophageal reflux or laryngopharyngeal reflux occurs. For example, some studies have shown that pepsin in saliva is a sensitive biomarker for the diagnosis of Gastroesophageal reflux disease (GERD) and laryngopharyngeal reflux (LPR).^149–152^ But the reported value of salivary pepsin, such as sensitivity and specificity, is highly variable among the research groups.^153^ Meta-analysis has investigated the diagnostic value of saliva pepsin in saliva for GERD [sensitivity: 0.60 (95% CI 0.41–0.76), specificity:0.71 (95% CI 0.51–0.86)] and LPR [sensitivity: 64% [95% CI 43–80%], specificity: 68% (95% CI 55–78%)].^149,154^ It may not be as helpful as previously believed. Differences in study design and enrollment criteria might be the source of heterogeneity. In addition, choosing a protocol for optimal saliva collection promotes the goal of using salivary pepsin as a biomarker for reflux in future studies.

Salivary amylase, including α-amylase and β-amylase, is the primary enzyme in saliva. It breaks down the large macromolecules, like sugars and amylopectin, and changes them into simpler components. Decreased salivary amylase is associated with increased obesity and inflammatory markers in children, such as MCP-1, TNF-α, IL-6, and CRP.^155^ Furthermore, decreased salivary amylase has been found to be strongly associated with OSCC and premalignant lesions, suggesting salivary amylase as a potential biomarker for the early detection of oral cancer.^156^ Besides, quantification of salivary amylase can be used as an evaluation index to assess salivary gland function in patients with oral cancer undergoing radiotherapy.^157^ Even a larger sample size is needed, Shah et al. have confirmed a considerable increase in salivary amylase levels in patients with diabetes, which means that salivary amylase can be a potential biomarker for the diagnosis and monitoring of diabetes.^158–160^

Other minor enzymes in salvia such as phosphatase and lysozymes help to catalyze the chemical reactions in the mouth, or act as the first defense of our body by performing their anti-bacterial, anti-viral, and anti-fungal properties, as well as killing other foreign agents.^161^ Lysozyme, derived from monocytes and macrophages, is widely presented in human tissues and secretions, including saliva, tears, and plasma. Currently, salivary lysozyme is expected to be a biomarker of stress. High pressure from examination or occupation can lead to lower concentrations of salivary lysozyme in students, nurses, or line workers.^162–164^ The same conclusion was obtained in more and more studies which demonstrated that salivary lysozyme was inversely associated with stress and suggested that stress influenced non-specific immunity. For oral diseases, active MMP-8 has been studied for point-of-care assay.^165,166^ It was strongly predictive for Stage IV periodontitis with a sensitivity of 89.7%, a specificity of 73.6%, and an AUC of 0.856.^165^ Besides, saliva levels of aspartate aminotransferase (AST) and proteinases are significantly associated with the intensity and extent of periodontal inflammation.^167,168^

All in all, saliva is rich in enzymes, which not only play an important role in the digestion of food, but also can be used as markers to diagnose or detect the occurrence and development of diseases. As a non-invasive detection method, salivary enzymes have wide application prospects.

### Immunoglobulin

Saliva contains a variety of immunoglobulins, including immunoglobulin A (IgA), IgG, IgM and IgE and so on. They play a significant role in the body and oral immunity. IgA, mainly secretory IgA (sIgA) produced by the plasma cells in salivary glands, is the most important and abundant antibody. Salivary IgA acts as the first line of defense against pathogenic bacteria. For example, numerous studies have indicated the protective role of salivary IgA against dental caries in both children and adults, by preventing the adhesion of bacteria to the tooth surface.^169–171^ Meanwhile, the levels of IgA in saliva are correlated with the risk of upper-respiratory tract infections (URTIs). Low salivary IgA levels can increase the frequency and duration of URTIs. Tsukinoki et al. that salivary IgA may help prevent SARS-CoV-2 infection.^172^ Calheira et al. standardized an immunoassay to detect salivary IgA antibody specific to Porphyromonas gingivalis antigens, to discriminate between individuals with and without leprosy.^173^ Leicht et al. considered that IgA in saliva might predict a change in lung infection status in patients with cystic fibrosis.^174^ In addition to salivary IgA, Riis et al. reported that the salivary Human Cytomegalovirus (HCMV) IgG test had 51% sensitivity and 97% specificity.^175^ With further technical development, HCMV IgG oral fluid testing might replace serum HCMV IgG measurement. Additionally, Kaplan et al. concluded that Immunoglobulin free light chains in saliva acted as a potential marker with the sensitivity of 89% and specificity of 80% for disease activity in multiple sclerosis.^176^ Although the contents of the Ig in saliva were lower than that in serum, the level of salivary Ig is sufficient for immunological diagnosis in several diseases.

### Glycoproteins

Glycoproteins are an important part of the salivary proteome and play a fundamental role in many biological processes.^177^ Accumulated evidence displayed that the aberrant glycoproteins in saliva were associated with tumor growth and progression as well as other diseases, making salivary glycoproteins promising diagnostic biomarkers.

In the study of diagnosis or prognosis in tumors, scholars have paved the way for salivary biomarkers application. Ragusa et al. have hydrolyzed the glycoproteins in saliva and quantified glycosylation levels. They found fucose levels higher in both cancer samples than in healthy controls, providing preliminary assessment for early diagnosis of tumor.^178^ Yang et al. have validated the differential expression level of galactosylated N/O-linked glycans in saliva in patients with breast cancer compared with healthy controls, promoting the study for early-stage diagnosis.^179^ Similarly, they also found alterations of salivary glycoprotein N-glycan profiles could be potential biomarkers distinguishing breast cancer or benign breast diseases and healthy subjects. This provided information on N-glycans during the development of breast cancer.^180^ It has also been reported that fucosylated N-/O-linked glycans in salivary proteins exhibited significantly differential expression levels in gastric cancer, facilitating the discovery of biomarkers for gastric cancer diagnosis.^181^ Zanotti et al. have observed that concentrations of saliva EGFR, a type of glycoprotein, were higher in oral cancer, leading to worse prognosis as well.^182^

In other diseases, glycoproteins in saliva can also exhibit potential as biomarkers while validation is needed in further clinical trials. A mucin-like glycoprotein called endogenous proteoglycan 4 (PRG4) in saliva samples was observed at elevated levels in SS patients, improving the understanding of the disease growth and treatment.^183^ Grant et al. have found that the best performing panels, including MMP9, S100A8, alpha-1-acid glycoprotein, and pyruvate kinase, could distinguish between periodontal health and disease states.^184^ In Alzheimer’s disease (AD), whether salivary lactoferrin could be the candidate biomarker reached inconsistent conclusions. A systematic review found that lactoferrin had the potential as future salivary biomarker for AD.^185^ However, Gleerup et al. have observed that salivary lactoferrin could not differentiate between neurodegenerative dementias.^186^ These findings indicated that heterogeneity of cohorts or medications leading to altered saliva production might impact the efficacy of potential biomarkers.

Overall, multiple glycoproteins in saliva have been investigated and may be candidates for future biomarkers in different diseases, while further validation should be carried out in larger and representative clinical cohorts.

### C-reaction protein (CRP)

CRP is a well-known sensitive inflammatory biomarker, usually detected in serum samples. As saliva sampling is non-invasive, simple, and patient-friendly, salivary CRP may be an alternative biomarker for clinical diagnosis.

In infectious diseases, CRP in saliva has been investigated to reflect the serum level. Jacobs et al. have reported that markers including salivary CRP may be indicative in the diagnosis of tuberculosis (TB), with a decent sensitivity of 78.1% and specificity of 83.3%.^187^ The salivary CRP level was reported much higher in patients (aged 2 to 17 years) with pneumonia than in healthy children (*P* < 0.001). Besides, saliva and serum CRP level was highly correlated in pediatric patients with pneumonia (*r* = 0.679; *P* < 0.001).^188^ Similarly, Lin et al. observed a correlation of CRP levels between serum and saliva samples over time in septic neonates.^189^ A higher salivary concentration of CRP was also observed in adult patients with sepsis compared to patients without sepsis, though without statistical difference (*P* > 0.05).^190^ The capacity of salivary CRP as a biomarker for sepsis both in neonates and adults exhibited differences, which needs further validation and mechanisms exploitation. Other than inflammation and infectious diseases, salivary CRP may also be potential biomarkers for other diseases such as acne vulgaris^191^ and Huntington’s disease.^192^ It indicated that inflammatory markers might also function in the physiological process of non-inflammatory diseases.

Salivary CRP is broadly detected via the enzyme-linked immunosorbent assay (ELISA) technique, which can be cumbersome and time-consuming. Therefore, many scholars have devoted themselves to developing efficient detection methods for clinical translation applications. For example, a photothermal biosensor for the measurement of CRP in saliva has been developed with rapid, simple, and minimally invasive characteristics.^193^ Many methods have also been investigated to improve analysis performance of detecting CRP in human saliva, such as the silicon nanowire luminescent sensor,^194^ a label-free and highly sensitive imaging sensor based on a plasmonic nano cup array,^195^ a label-free biosensor based on an electrolyte-gated organic thin-film transistor (EGOTFT),^196^ electrochemical methods,^197^ etc. However, there’s still a long way to go for the clinical application of these novel methods due to sample heterogeneity and small sample size.

### Hormones

**Fig. 2:**
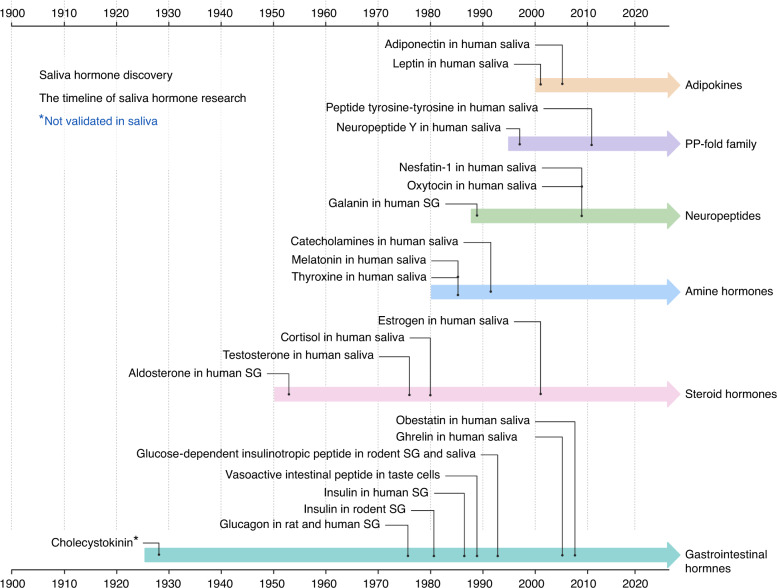
The timeline of salivary hormones. The time was determined by the publishing year of the oldest and most relevant papers. Not all hormones were included since there was no evidence showing the existence of them in saliva or the poor correlation of the concentration between saliva and target organ, such as follicle-stimulating hormone (FSH). The figure is created with BioRender.com

Steroids, non-steroids, peptides, and protein hormones can be detected in saliva. Nevertheless, some hormones are of little use for saliva testing, such as dehydroepiandrosterone sulfate, thyroxine, and pituitary hormones.^12^ The research timeline of valuable salivary hormones was extracted from published papers,^12,198–212^ displayed in Fig. 2.

Cortisol is a hormone secreted by the adrenal glands that can regulate the metabolism of sugar and is distributed in various body fluids, including saliva, urine, and serum. Cortisol has been studied as a biomarker of diverse diseases. Gozansky et al. have found that salivary cortisol is better than serum total cortisol for evaluation of dynamic hypothalamic-pituitary-adrenal (HPA) axis activity, because more physiologically relevant data can be obtained from salivary cortisol.^213^ The HPA axis is the main neuroendocrine axis that regulates mammalian stress responses.^214^ Dysfunction of the HPA axis is associated with mental and functional problems, such as anxiety, insomnia, depression, bipolar disorder, affective disorder, cognition impairment, fibromyalgia (FM), allergic bowel syndrome, and alcoholism. HPA axis reacts to the stressor by secreting cortisol. Salivary cortisol, as a stress marker, has become a hot spot for research in recent years.^164,215^ A close relationship was found in patients with FM between salivary cortisol levels and the disease process. Compared with healthy individuals, increased salivary cortisol levels were detected in initial stages of FM, while decreased levels were detected in those with a longer duration of disease.^216^ Cortisol was reported to be a useful salivary biomarker of non-motor symptoms in patients with PD, since approximately higher levels of cortisol were identified in PD compared to the healthy controls.^138^ Furthermore, salivary cortisol can be used for differential diagnosing of Cushing’s syndrome,^217,218^ monitoring the state of oxidative stress,^219^ and recognizing inflammatory periodontal diseases (IPD).^220^ Cortisol testing often needs to be conducted at midnight, which is not always possible. More importantly, venous blood collection would cause the fluctuation of cortisol levels, which decrease the accuracy of evaluation. Saliva collection by the patients themselves is convenient and accurate. It has been utilized in clinical diagnosis.

Oxytocin, a neuro-hormone produced by the hypothalamus and finally released from the posterior hypophysis,^221^ is distributed in the periphery and central nervous system.^222^ Over the past decades, oxytocin has been extensively studied due to its important function in reproductive physiology, affective processes, and social behaviors, including stimulating the milk production in mammary glands, promoting the uterine smooth muscle contraction during labor, regulating parental nurturing behaviors and empathy-related behaviors.^221,223,224^ In addition, oxytocin was reported to be associated with pain relief in aging.^222^ A negative correlation was also found between oxytocin levels and depression/anxiety.^225^ Though saliva as an easy-access and low-invasive sample can be a good choice for oxytocin analysis, salivary oxytocin levels represent only 5%-10% of blood oxytocin levels, making the measurement of salivary oxytocin very difficult.^226^ The application of salivary oxytocin is limited by the means of detection. With the rapid advancement of science and technology, salivary oxytocin holds a great prospect for research and applications.

Melatonin, a neurohormone produced by the pineal gland, can regulate circadian rhythms (sleep-wake rhythm, neuroendocrine rhythms, and body temperature cycles).^227^ Melatonin is presented in various body fluids, including blood, saliva, and cerebrospinal fluid. The level of melatonin in the saliva is about 30% of that in blood, recognized to present the free, unbound plasma melatonin levels.^228^ Like blood melatonin, salivary melatonin is an indicator of the circadian phase.^228^ Besides, the detection and quantification of daytime salivary melatonin content can positively reflect the levels of some depression-related inflammatory markers, such as CCL2/MCP-1, CCL3/MPI-1α, and VEGF-A.^229^ Yvan et al. has found a positive correlation between salivary melatonin levels and urinary excretion of 6-sulphatoxymelaton in children. This suggested salivary melatonin a potential marker for the diagnosis of chronobiological disorders in prepubertal children.^230^ Further, melatonin has protective, oncostatic, and antioxidant properties.^231^ Salivary melatonin levels in patients with lung cancer and prostate cancer were lower compared to those in controls.^232,233^

Sex hormones can also be detected in saliva. Mitova et al. have proved that saliva is an appropriate medium for studying the quantities of sex hormones.^234^ It has been reported that there was a high linear correlation between salivary and plasma for 17a-hydroxyprogesterone (17-OHP), progesterone (P), androstenedione (A4), and cortisol (F).^235^ In addition, salivary steroids stand for only the free, bioactive fraction of steroids circulating in blood.^236^ The detection of salivary cortisol at midnight is routinely used to screen for Cushing’s syndrome.^237^ Also, salivary testosterone was correlated significantly with free serum testosterone in adult men and women by an improved liquid chromatography/tandem mass spectrometry or mass spectrometry (LC-MS/MS) method.^238^ Saliva testosterone may be a reliable alternative to serum testosterone in the diagnosis of androgen disorders and monitoring the disease status in clinical research. The amount of asprosin hormone, reported for the first time by Ugur et al., was higher in saliva than in blood. The increased level of asprosin in saliva correlates with increased BMI.^239^ The properties have made salivary hormone measurement beneficial for various disciplines like psychology, endocrinology, and pediatrics to assess endocrine sex-related hormone levels.^236^

Multiple platforms have been established to measure the concentration of the hormones in the saliva. Arevalo et al. employed an electrochemical bioplatform for simultaneously determining four fertility-related target hormones, such as estradiol (E2), progesterone (P4), luteinizing hormone (LH), and prolactin (PRL).^240^ Xu et al. described a method of LC/MS for the simultaneous quantification of seven trace steroid hormones in human saliva and showed excellent specificity and sensitivity.^241^ With the rapid development of technology, the detection of salivary hormones has a wide application prospect in the diagnosis of diseases and in monitoring the disease status.

### Metabolomic biomarkers

Saliva metabolome has been largely understudied compared to genome, transcriptome, and proteome. Researchers analyzed the metabolomes of 16 healthy adults.^242^ They were able to quantify or identify 308 salivary metabolites in human saliva. Based on the identified metabolites, researchers also explored the potential use of them in distinguishing disease status. Zhou et al. found that metabolomic biomarkers could assist in diagnosis and monitoring of orthodontically induced external apical root resorption (OIEARR).^243^ Some scholars also agreed with the point that saliva metabolome was meaningful for oral diseases. They found that saliva represented as metabolomic fingerprints for patients with periodontitis.^244^ For more severe oral disease, such as OSCC, saliva metabolome also showed its promising result. In a Japanese cohort, 25 metabolites were revealed as potential biomarkers to discriminate OSCC patients and healthy controls.^245^ Similar as the interests about epigenetic change brought by environmental factors, a study also paid attention to metabolomic profile alteration under different environment.^246^ They concluded that the metabolomic features could be used in identification and measurement of exposures to mobile source air toxics.

### Exosome biomarkers

Saliva has less complicated components than blood, and the salivary exosome biomarkers are enriched 1000-fold lower than that of biomarkers in blood.^247^ Exosomes contain abundant proteins, mRNA, microRNAs, cytokines, and transcription factor receptors,^248^ which can be promising in cancer diagnosis, aging research,^249^ and many other diseases.^250–252^ Using saliva exosomes avoids potential contaminants or confusing substances, such as food debris, thus benefiting biomarker discovery. Some differences in substances were only present in salivary exosomes, not whole saliva.^111^ For instance, saliva membrane-based markers CD9 and CD81 were expressed differently in oral disease patients and healthy individuals.^253,254^ It is reported that microRNAs detected in saliva and serum are mostly enriched in exosomes.^255^ Saliva exosomes were mainly secreted by salivary glands, with part of them from oral colonization flora. By shotgun mass spectroscopy analysis, novel biomarkers of salivary exosomes in patients with inflammatory bowel disease (IBD) and healthy controls for IBD were revealed.^256^ The study found that proteasome subunit alpha type 7 (PSMA7) showed apparent differences between patients with IBD and healthy controls. In IBD, salivary exosomal PSMA7 plays a role in identifying IBD with healthy controls.^256^ Sun et al. confirmed the existence of BPIFA1, CRNN, MUC5B, and IQGAP either in salivary microvesicles or in exosomes between normal subjects and lung cancer patients.^257^ Additionally, neurodegenerative diseases were given much attention for saliva exosome biomarker discovery.^258^

There have been reviews summarizing salivary exosomal biomarkers in cancer,^259–263^ neurodegenerative diseases,^264^ mental diseases,^265^ systematic disease,^266^ wound healing^267,268^ and isolation methods.^269,270^ Research articles emphasize protein biomarkers^250,257,271–275^ and RNA markers.^276–279^ The methodology of salivary exosomes mainly focuses on isolation, quantification, and characterization.^280,281^ Some point-of-care testing kits were also invented. Detecting exosomal miRNA in saliva was useful for the diagnosis of lung cancer.^282^

A few studies aimed at resolving the regulating mechanism of salivary exosomes. Salivary exosomes from mice with pancreatic ductal adenocarcinoma (PDAC) suppress the tumor-killing capacity of nature killing (NK) cells.^275^ CD9 and CD81 participate in the functional regulation of cell adhesion and migration. Decreased expression of these two markers benefits the metastasis of tumor cells.^254^ On the contrary, overexpressed miR-24–3p promotes the propagation of tumor cells by interacting directly with the PER1 gene.^277^ Programmed death-ligand 1 (PD-L1) mRNA in salivary exosomes inhibits the damage to the inflammatory system.^283^

### Saliva collection and preservation

There are two types of whole saliva distinguished by the method of collection, unstimulated whole saliva (USWS) and stimulated whole saliva (SWS). Passive drooling and spitting method are primarily used in collecting USWS. For the collection of SWS, chewing different things (like natural gum) have been used. Recent developments in standardized saliva collection devices allow safe, simple, and convenient collection of samples from patients. For a more detailed overview of saliva collection methods and devices, the reader is referred to previous reviews on this subject.^284–287^ Briefly, the Super·Sal™ (Oasis Diagnostic) and Versi·Sal™ (Oasis Diagnostic) devices sample unstimulated whole saliva by passive drooling, while the Salivette® (Sarstedt) and SalivaBio Oral Swab (SOS, Salimetrics) can sample unstimulated saliva from specific regions or stimulated saliva by simply moving it in the oral cavity.^285^ With the availability of these new technologies, researchers have focused on the effect of saliva collection methods and devices on different salivary biomarkers. We reviewed those advances to contribute insight into saliva sampling for further study design and clinical application.

The influence of saliva collection methods and devices on different types of salivary biomarkers are summarized in Table 3. Although different saliva collection methods yielded similar proteome coverage, the specific protein types and concentrations observed depended on the collection approach, because of different salivary flow rates and protein secretion rates.^288–293^ Rigid criteria for saliva collection may not be required in salivary nucleic acid analysis. Several studies have revealed that the method of saliva collection has a minimal impact on nucleic acid analysis, including exRNA recovery, mRNA expression, gDNA profiles, DNA quality and quantity, DNA methylation levels and epigenetic factors.^292,294–296^

**Table 3:** The influence of saliva collection methods on salivary biomarkers

Comparative saliva collection methods	Main results	Reference
Drooling, SOS, Salivette®, Synthetic, and Greiner Bio-One Saliva Collection System (SCS)	SOS yielded lower concentrations of myoglobin and CRP the Salivette® Cotton and Synthetic swab yielded lower myoglobin and IgE concentrations	^288^
Drooling, paraffin gum, and Salivette®	Saliva volume: paraffin gum > Salivette® > drooling protein concentrations: no significant differences proteome coverage: about 160 proteins of each approach specific proteins depended on the collection approach: observed	^289^
Stimulated by Salivette®, Parafilm®, chewing gum, and unstimulated from spit with and without fluid accumulation	Flow rate: chewing gum > Salivette® = Parafilm® > unstimulated total protein: chewing gum > Parafilm® > others nitrite secretion: chewing gum > others total antioxidant capacity: chewing gum > others alpha-amylase concentration: unstimulated without saliva accumulation < stimulated	^290^
Before and after oral hygiene	Oral hygiene decreased salivary flow, reduced the secretion rate of total protein and increased sAA	^290^
Salivette® and spiting	Visual similar MS spectra for each individual	^291^
RNAPro·SAL™ and standard clinical collection process	Similar protein and exRNA recovery and stability	^292^
Passive drooling, Pure·SAL™, and RNAPro·SAL™	Pure·Sal™ and RNAPro·Sal™ resulted in much clearer protein spots in comparison to passive drooling	^293^
Spitting and drooling	No signifcant diferences in bacterial profles	^294^
Spitting and drooling	No significant influence on periodontium-associated mRNA expression, DNA methylation levels and epigenetic factors	^293^
GeneFiX Saliva DNA Collection Kit	Drinking or eating right before collection does not influence the quantity or quality of the isolated DNA	^296^
Salivette® with and without stimulation	Compared with unstimulated saliva, stimulated saliva had decreased glucose levels and increased salivary flow.	^303^

In addition to the standardized collection method, an appropriate storage condition is probably another key factor for the successful analysis of saliva constituents. Human salivary exosomes remained undestroyed without the existence of protease inhibitor, under different storage temperatures.^297^ However, owing to the interference of complicated saliva components, in particular bacteria and enzymes, proper saliva storage is mandatory. Generally, the saliva collection tube should be properly sealed and placed upright.^298^ The saliva sample should be kept between 2 and 8 °C in cold packs or containers with ice, and transported to the processing laboratory as soon as possible within 24 h to a maximum of 48 h. After arrival at the laboratory, the saliva sample should be processed immediately, otherwise directly stored at -80 °C.^299,300^ Alternatively, when using commercial collection kits, transportation and storage requirements may differ, so it’s important to ensure that all instructions for manufacturers are followed.^298^ Moreover, there are also differences in the recommended preservation condition and time for specifically interested biomarkers (Table 4).

**Table 4:** Stabilities of salivary biomarkers on different preservation conditions

Biomarkers	Collection methods	Preservatives	Temperature	Stable Time	Reference
Total protein	Sublingual cotton roll	None	RT	≥ 24 hours	^304^
SAA, UA, total protein and low-molecular-weight antioxidants	Saliva Collection System	None	-20 °C	≥ 2 weeks	^305^
IgA and lysozyme	unstimulated saliva	None	-30 °C	≥ 3 months	^306^
Cortisol	Salivette®	NaN3	18°C	≥ 3 weeks	^307^
		None	5°C	≥ 3 months	^308^
			-20°C/-80°C	≥ 1 year	
Total RNA	Drooling	RNAlater	RT	≥ 7 days	^309^
			-80°C	≥ 4 weeks	
Metabolomics	Drooling	None	RT/4°C	≥ 6 hours	^310^
			-20°C	≥ 4 weeks	
		NaN3	25°C	≥ 8 hours	
			4°C	≥ 48 hours	

## Conclusion

In this review, the updated research about salivary biomarkers was discussed. RNA biomarkers were most widely studied in all kinds of diseases. However, in our opinion, hormones were the most promising markers in clinical diagnostics. Future studies need to be conducted to overcome the fluctuation caused by the circadian rhythm and convenient wearable or portable devices for monitoring the dynamic changes in patients. This will benefit a lot for patients who need to be tested multiple times and are hard to obtain blood or other types of body fluids.

## Supplementary information

Supplementary table 1

Supplementary Figure 1
