# Supplementary material for: Promising applications of human-derived saliva biomarker testing in clinical diagnostics
Source: Int J Oral Sci. 2023 Jan 4;15:2. doi: 10.1038/s41368-022-00209-w (PMC9810734; doi:10.1038/s41368-022-00209-w)
Supplement: Supplementary file 1 — Supplementary table 1 [file 41368_2022_209_MOESM1_ESM.docx]

**Supplementary table 1.** The summary of studies about saliva microRNAs in different kinds of diseases.

| **Type of disease** | **Patient(n)** | **Control**  **(n)** | **Marker sets** | **Sensitivity (%)** | **Specifcity (%)** | **Detection method** | **Year of**  **publication** | **References** |
| --- | --- | --- | --- | --- | --- | --- | --- | --- |
| Oral squamous cell carcinoma | 50 | 50 | miR-200a | - | -AUC 0.65 | RT-PCR | 2009 | ^303^ |
|  | 50 | 50 | miR-125a | - | -AUC 0.62 | RT-PCR | 2009 | ^303^ |
|  | 50 | 50 | miR-200a+miR-125a | - | -AUC 0.66 | RT-PCR | 2009 | ^303^ |
|  | 21 | 43 | miR-31 | 80.0 | 65.0 | RT-PCR | 2011 | ^304^ |
|  | 45 | 34 | miR-31 | 80.0 | 68.0 | RT-PCR | 2012 | ^305^ |
|  | 20 | 20 | miR-21 | 60.0 | 90.0 | RT-PCR | 2015 | ^306^ |
|  | 20 | 20 | miR-145 | 60.0 | 70.0 | RT-PCR | 2015 | ^306^ |
|  | 20 | 20 | miR-184 | 80.0 | 75.0 | RT-PCR | 2015 | ^306^ |
|  | 17 | 17 | miR-27b | 85.7 | 100.0 | RT-PCR | 2017 | ^307^ |
|  | 17 | 17 | miR-136 | 88.9 | 100.0 | RT-PCR | 2017 | ^307^ |
|  | 18 | 18 | miR-146a-5p | 78.0 | 85.0 | RT-PCR | 2017 | ^308^ |
|  | 21 | 11 | miR-512-3p | 80.0 | 70.0 | RT-PCR | 2018 | ^309^ |
|  | 21 | 11 | miR-412-3p | 80.0 | 95.0 | RT-PCR | 2018 | ^309^ |
|  | 25 | 25 | miR-139-5p | 73.9 | 85.0 | RT-PCR | 2016 | ^106^ |
|  | 53 | 136 | miR-24-3p + miR-21-5p + miR-99a-5p + let-7c-5p + miR-100-5p | - | -AUC 0.867 | RT-PCR | 2019 | ^310^ |
|  | 45 | 10 | miR-24-3p | 80.0 | 73.8 | RT-PCR | 2019 | ^311^ |
|  | 89 | 58 | miR-106b-5p, miR-423-5p and miR-193b-3p | - | AUC  0.98 | Microarray  RT-PCR | 2021 | ^312^ |
| Oral squamous cell carcinoma- cervical lymph node metastasis | 68 | 62 | miR-21-5p | 42.6 | 90.3 | RT-PCR | 2021 | ^313^ |
|  | 68 | 62 | miR-21-3p | 60.3 | 83.9 | RT-PCR | 2021 | ^313^ |
| Oral potentially malignant disorders | 36 | 36 | miRNA-21 | 0.69 | 0.66 | RT-PCR | 2020 | ^314^ |
| Head and neck squamous cell carcinoma | 56 | 56 | miR-9, miR-134 and miR-191 | - | - | RT-PCR | 2014 | ^315^ |
|  | 47 | 113 | miRNA-9, -127, -134, -191, -222, and -455 | 60.0 | 94.0 | RT-PCR | 2017 | ^316^ |
|  | 54 | 113 | miRNA-9-134, -210, -455, and -196b | 65.0 | 95.0 | RT-PCR | 2017 | ^316^ |
|  | 150 | 80 | miR-let-7a-5p | 0.70 | 0.80 | RT-PCR | 2020 | ^317^ |
|  | 150 | 80 | miR-3928 | 0.75 | 0.80 | RT-PCR | 2020 | ^317^ |
|  | 108 | 108 | miR-122-5p, miR-92a-3p, miR-124-3p, and miR-146a-5p | - | - | RT-PCR | 2018 | ^318^ |
| Nasopharyngeal carcinoma | 22 | 25 | 12 miRNAs | 100.0 | 96.0 | Microarray  RT-PCR | 2018 | ^319^ |
| Esophagus | 32 | 16 | miR-21 | 84.4 | 62.5 | RT-PCR | 2012 | ^320^ |
|  | 39 | 19 | miR-10b | 89.7 | 57.9 | RT-PCR | 2013 | ^321^ |
|  | 39 | 19 | miR-144 | 92.3 | 47.4 | RT-PCR | 2013 | ^321^ |
|  | 39 | 19 | miR-451 | 84.6 | 57.9 | RT-PCR | 2013 | ^321^ |
|  | - | - | miR-196a | - | - | RT-PCR | 2017 | ^322^ |
|  | 72 | 50 | miR-1246 | 83.3 | 66.0 | RT-PCR | 2021 | ^323^ |
| Parotid gland tumors | 38 | 29 | 4 miRNAs | 69.0 | 95.0 | RT-PCR | 2013 | ^324^ |
|  | 78 | 38 | hsa-miR-211+hsa-miR-1233 | 91.0 | 86.0 | RT-PCR | 2015 | ^325^ |
| Pancreas | 40 | 60 | miR-3679-5p+ miR-940 | 70.0 | 70.0 | RT-PCR | 2015 | ^326^ |
|  | 7 | 4 | miR-21 | 71.4 | 100.0 | RT-PCR | 2015 | ^327^ |
|  | 7 | 4 | miR-23a | 85.7 | 100.0 | RT-PCR | 2015 | ^327^ |
|  | 7 | 4 | miR-23b | 85.7 | 100.0 | RT-PCR | 2015 | ^327^ |
|  | 7 | 4 | miR-29c | 57.0 | 100.0 | RT-PCR | 2015 | ^327^ |
|  | 41 | 30 | miR-1246 | 91.0 | 26.7 | RT-PCR | 2020 | ^328^ |
| Pancreatobiliary tract cancer | 12 | 13 | miR-1246 | 66.7 | 100.0 | RT-PCR | 2016 | ^329^ |
|  | 12 | 13 | miR-4644 | 75.0 | 76.9 | RT-PCR | 2016 | ^329^ |
|  | 12 | 13 | miR-1246+miR-4644 | 83.3 | 92.3 | RT-PCR | 2016 | ^329^ |
| Hand, Foot, and Mouth Disease | - | - | 6 miRNAs | 78.3-100.0 | 76.0-88.9 | RT-PCR | 2018 | ^111^ |
|  | - | - | 4 miRNAs | 68.8-100.0 | 69.6-87.5 | RT-PCR | 2018 | ^111^ |
| Autism spectrum disorder | 24 | 21 | 14 miRNAs | 81.0 | 87.5 | RNA-Seq | 2016 | ^104^ |
|  | - | - | miR-28–3p+miR-151-a-3p+miR-148a-5p+miR-125b-2–3p | 89.20 | 32.0 | RNA-Seq | 2020 | ^105^ |
|  | 39 | 41 | 5 miRNAs | 93.1 | 54.6 | RT-PCR | 2020 | ^106^ |
| Alcohol dependence | 60 | 60 | 10 miRNAs | 65.1-79.2 | 64.8-89.0 | RNA-Seq | 2019 | ^107^ |
| Colorectal | 34 | 34 | miR-21 | 97.0 | 91.0 | RT-PCR | 2016 | ^330^ |
|  | 51 | 37 | miR-186-5p | 64.7 | 54.1 | RT-PCR | 2019 | ^331^ |
|  | 51 | 37 | miR-29a-3p | 64.7 | 56.8 | RT-PCR | 2019 | ^331^ |
|  | 51 | 37 | miR-29c-3p | 64.7 | 62.2 | RT-PCR | 2019 | ^331^ |
|  | 51 | 37 | miR-766-3p | 64.0 | 62.2 | RT-PCR | 2019 | ^331^ |
|  | 51 | 37 | miR-491-5p | 70.6 | 50.0 | RT-PCR | 2019 | ^331^ |
| Periodontitis | 120 | 80 | miR-23a | 81.7 | 83.8 | RT-PCR | 2021 | ^332^ |
|  | 120 | 80 | miR-146a | 82.5 | 86.3 | RT-PCR | 2021 | ^332^ |
|  | 10 | 10 | hsa-miR-140-5p, hsa-miR-146a-5p, hsa-miR-628-5p | - | -AUC 0.93-1 | RT-PCR | 2020 | ^252^ |
| Sjögren's syndrome | 24 | 16 | miR-17-5p+let-7i-5p | - | - | RT-PCR | 2020 | ^110^ |
| Peri-implantitis | 4 | 4 | miR-4484 | - | - | RNA-Seq | 2021 | ^333^ |
| Concussion | 106 | 50 | a combined panel of 14 sncRNAs | - | -AUC 0.93-0.96 | RNA-Seq | 2021 | ^109^ |
