# Supplementary figures and images for: Promising applications of human-derived saliva biomarker testing in clinical diagnostics
Source: Int J Oral Sci. 2023 Jan 4;15:2. doi: 10.1038/s41368-022-00209-w (PMC9810734; doi:10.1038/s41368-022-00209-w)

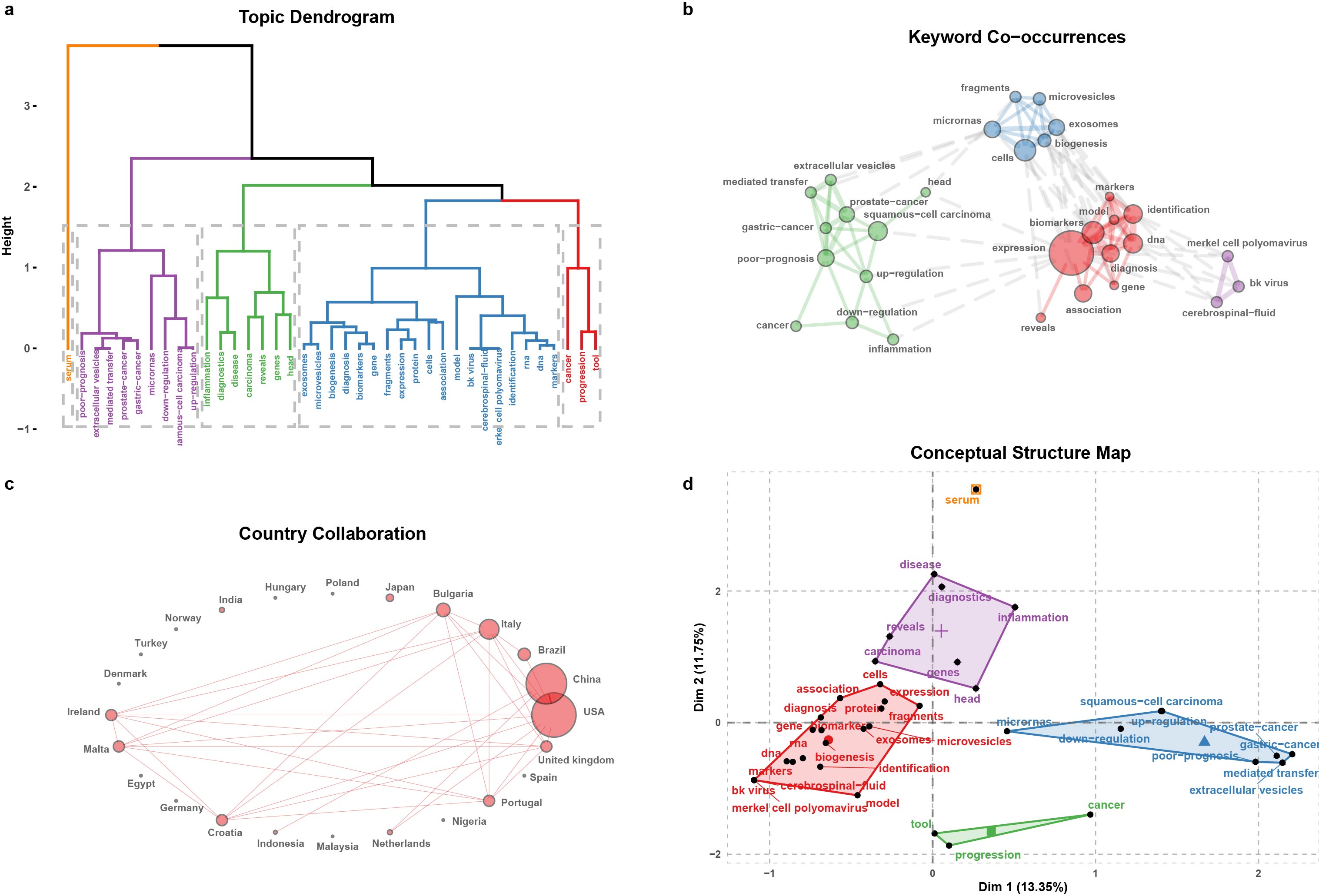

Supplement: Supplementary file 2 — Supplementary Figure 1 [file 41368_2022_209_MOESM2_ESM.jpg]
